# Somatic *Trp53* mutations differentially drive breast cancer and evolution of metastases

**DOI:** 10.1038/s41467-018-06146-9

**Published:** 2018-09-27

**Authors:** Yun Zhang, Shunbin Xiong, Bin Liu, Vinod Pant, Francis Celii, Gilda Chau, Ana C. Elizondo-Fraire, Peirong Yang, Mingjian James You, Adel K. El-Naggar, Nicholas E. Navin, Guillermina Lozano

**Affiliations:** 1 Department of Genetics, University of Texas MD Anderson Cancer Center, Houston, TX 77030 USA; 2 Department of Hematopathology, University of Texas MD Anderson Cancer Center, Houston, TX 77030 USA; 3 Department of Pathology, University of Texas MD Anderson Cancer Center, Houston, TX 77030 USA; 4 Department of Pharmaceutical and Environmental Health Sciences, College of Pharmacy, Texas Southern University, Houston, TX 77004 USA

## Abstract

*TP53* mutations are the most frequent genetic alterations in breast cancer and are associated with more aggressive disease and worse overall survival. We have created two conditional mutant *Trp53* alleles in the mouse that allow expression of *Trp53R172H* or *Trp53R245W* missense mutations in single cells surrounded by a normal stroma and immune system. Mice with *Trp53* mutations in a few breast epithelial cells develop breast cancers with high similarity to human breast cancer including triple negative. p53R245W tumors are the most aggressive and exhibit metastases to lung and liver. Development of p53R172H breast tumors with some metastases requires additional hits. Sequencing of primary tumors and metastases shows p53R245W drives a parallel evolutionary pattern of metastases. These in vivo models most closely simulate the genesis of human breast cancer and will thus be invaluable in testing novel therapeutic options.

## Abstract (web summary)

Mutations in *TP53* gene are very common in cancer development. Here the authors take advantage of murine models to show that somatic *Trp53* mutations differentially drive breast cancer and evolution of metastases.

## Introduction

Breast cancer is a heterogeneous disease generally comprised of five molecular subtypes: luminal A, luminal B, normal-like, HER-2 enriched, and basal-like^1,2^, with the last two subtypes having poorer prognosis and the highest *TP53* mutation frequency^2^. In particular, the majority of these mutations occur on three arginines, R175, R248, and R273, in the DNA binding domain of *TP53*, with R248 being the top mutational hotspot in breast cancer^2^. To investigate the role of *Trp53* mutations in breast cancer, a number of mouse models have been established. For example, mice containing germline *Trp53* mutations model Li-Fraumeni Syndrome (LFS)^3^, an inherited cancer syndrome associated with germline *Trp53* mutations, and develop highly metastatic mammary tumors in a Balb/C background. On the other hand, *TP53* mutations are largely spontaneous and somatic^4^. The heretofore published *Trp53* alleles, which allow spontaneous expression of mutant Trp53, have indeed played an important part in advancing our understanding of somatic p53 function in breast cancer, but exhibit critical caveats that undermine their clinical faithfulness. For example, a number of transgenic lines, which overexpress mutant Trp53 in mouse mammary glands, have been particularly useful for understanding the effect of different forms of mutant Trp53 in mammary development and tumorigenesis, as mutant *Trp53* transgenes are often driven by mammary epithelium specific promoters^5,6^. However, such models usually rely on random integration of multiple copies of *Trp53*, which are present together with two endogenous WT *Trp53* alleles, and therefore the expression levels and activities of the *Trp53* transgene may not accurately model endogenous mutated *TP53* alleles observed in human tumors. In addition, two knock-in conditional *Trp53R172H* (corresponding to human TP53R175H) and *Trp53R270H* (corresponding to human TP53R273H) alleles (referred to as *Trp53*^*LSL-mut*^), in which a *loxP* flanked STOP cassette was inserted into the first intron to block mutant Trp53 expression, have been utilized to study different types of cancers including breast cancer^7–9^. However, it is important to note that insertion of the STOP cassette makes the whole *Trp53*^*LSL-mut/+*^ mouse heterozygous for *Trp53*, which complicates the interpretation of the tumor phenotypes since studies have shown that impaired *p53* status in tumor microenvironment facilitates tumor development^10,11^.

Therefore, better models that faithfully mimic human sporadic breast cancer involving somatic *TP53* mutations are needed. These models will be critical for determining if and how expression of spontaneous p53 mutant alone in a limited number of epithelial cells can drive breast tumorigenesis, whether different hot spot *p53* mutations have the same potency, and whether these breast tumors could, at least partially, recapitulate characteristics of spontaneous human breast cancers. In addition, the emerging evidence for inter-patient heterogeneity and the notion of personalized therapy urge an understanding of the breast cancers specifically driven by *p53* mutations. It will be important to understand the heterogeneity and mutational evolution of breast tumors initiated by somatic *p53* mutations, which may enable tailoring of therapeutic strategies to the fundamental molecular lesions driving a particular tumor.

In this study, we generate two conditional *Trp53* alleles, which allow us to focally convert wild type (WT) p53 into a mutant, p53R172H or p53R245W (corresponding to human p53R175H and p53R248W, respectively), in mammary epithelial cells while maintaining WT p53 in the rest of the mouse. We find that p53R245W is more potent in driving mammary tumorigenesis and metastasis than p53R172H. In addition, multi-region whole exome sequencing reveals inter- and intra-tumor heterogeneity, as well as a parallel mutational evolutionary pattern in p53R245W-driven primary tumors and associated metastases.

## Results

### Generation of the *Trp53*^*wm-R172H*^ and *Trp53*^*wm-R245W*^ alleles

**Fig. 1:**
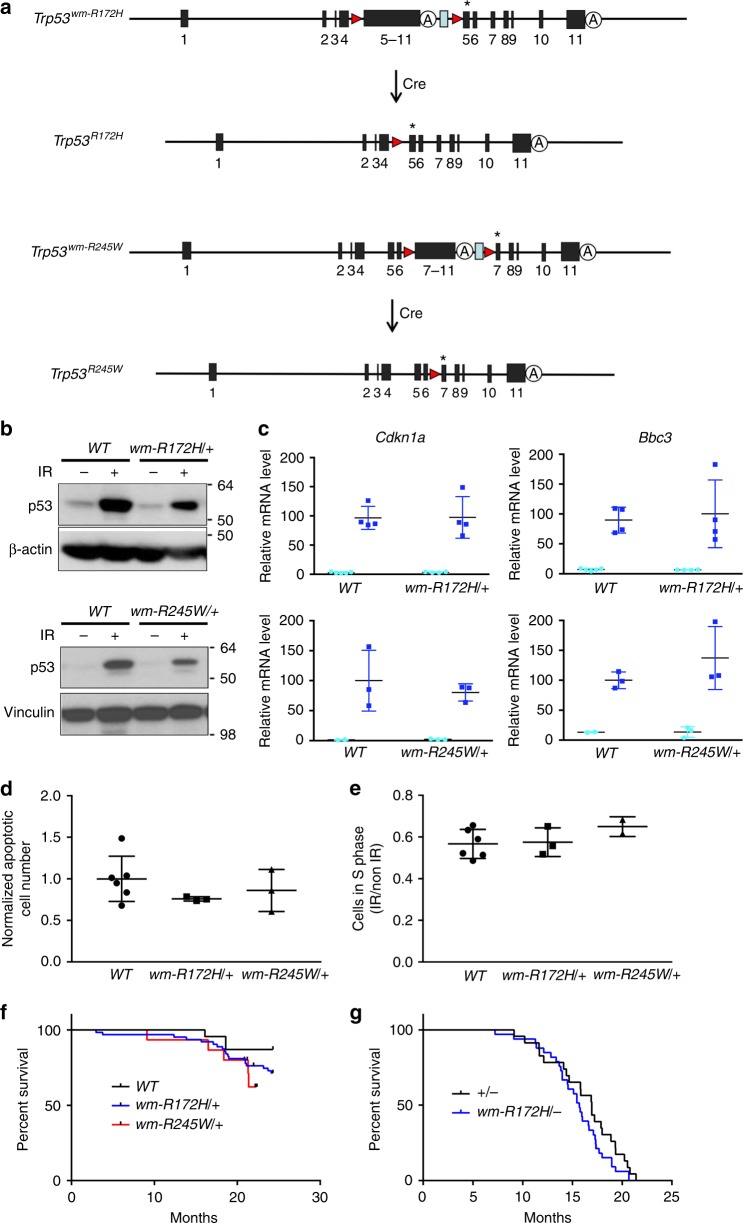
Generation and characterization of the *Trp53*^*wm-R172H*^ and *Trp53*^*wm-R245W*^ alleles. **a** Schematic representation of the *Trp53*^*wm-R172H*^ and *Trp53*^*wm-R245W*^ alleles. The cre-*loxP*-mediated strategy was used to generate the *Trp53*^*R172H*^ and *Trp53*^*R245W*^ alleles. Red triangle *loxP* site; blue rectangle *Frt* site left after removing the selection markers (see Supplementary Fig. 1); A, *Trp53* native polyadenylation signaling sequence; * 515G->C mutation was introduced in exon5 for the *Trp53*^*wm-R172H*^ allele, and 733 C→T and 735 C→G mutations were introduced in exon 7 for the *Trp53*^*wm-R245W*^ allele. **b** Western blot analysis for p53 protein levels in thymuses of various *Trp53* genotypes (*WT, wm-R172H/+* and *wm-R245W/+*). IR, γ-radiation. **c** Real-time reverse transcription PCR analysis for *Cdkn1a* and *Bbc3* mRNAs in thymuses of various *Trp53* genotypes (*WT, wm-R172H/+* and *wm-R245W/+*). Light blue circles, mRNA level of *Cdkn1a* or *Bbc3* in non-irradiated thymuses; Dark blue squares, mRNA level of *Cdkn1a* or *Bbc3* in irradiated thymuses. Error bars, s.d. **d** Quantification of the normalized number of γ-radiation-induced apoptotic thymocytes of various *Trp53* genotypes (*WT, wm-R172H/+* and *wm-R245W/+*), following annexin V staining and FACS analysis. Error bars, s.d. **e** Quantification of cells in S phase for γ-irradiated and non-irradiated MEFs of various *Trp53* genotypes (*WT, wm-R172H/+* and *wm-R245W/+*). Error bars, s.d. **f** Kaplan–Meier survival curves of *Trp53 WT* (*n* = 23), *Trp53*^*wm-R172H/+*^ (*n* = 42) and *Trp53*^*wm-R245W/+*^ (*n* = 15) mice. **g** Kaplan–Meier survival curves of *Trp53*^*+/−*^ *n* = 23) and *Trp53*^*wm-R172H/-*^ (*n* = 33) mice. In **c**–**g**, no statistical differences were observed between samples with different *Trp53* genotypes (*WT*, *wm-R172H/+* and *wm-R245W/+*)

To study how a somatic *p53* missense mutation drives breast cancer development, we generated two conditional *Trp53* alleles, which allowed us to convert WT *Trp53* to either *Trp53R172H* or *Trp53R245W* and to generate a *Trp53* missense mutation in a single cell or tissue in vivo. Briefly, a partial WT *Trp53* cDNA encoding exons 5–11 or exons 7–11 flanked by *loxP* sites was inserted into intron 4 or 6 of the endogenous locus of *Trp53* carrying a specific mutation in exon 5 or 7, respectively (Fig. 1a and Supplementary Fig. 1a, b). These alleles named *Trp53*^*wm-R172H*^ or *Trp53*^*wm-R245W*^ (wm refers to wild type to mutant) express WT p53 initially. Cre recombinase excises the wildtype cDNA to generate the mutant *Trp53*^*R172H*^ and *Trp53*^*R245W*^ alleles (Fig. 1a). The *Trp53*^*R172H*^ allele is identical to a previous germline mutant *Trp53* allele^12^, and germline *Trp53*^*R245W*^ mice are tumor-prone (unpublished data). Sequencing of the alleles ensured absence of other alterations (For details, please refer to the Methods section).

The *Trp53*^*wm-R172H*^ and *Trp53*^*wm-R245W*^ alleles express a full-length p53 protein in heterozygous mice, which is stable in response to γ-radiation (Fig. 1b and Supplementary Fig. 2), and can transcriptionally activate canonical p53 targets, *Cdkn1a* (*p21*) and *Bbc3* (*Puma*), similar to WT p53 (Fig. 1c). In addition, thymuses of *Trp53*^*wm-R172H/+*^ and *Trp53*^*wm-R245W/+*^ mice showed similar levels of apoptosis in response to γ-radiation (Fig. 1d) as WT mice. No differences in cell cycle arrest were observed between irradiated cultures of *WT*, *Trp53*^*wm-R172H/+*^ and *Trp53*^*wm-R245W/+*^ mouse embryo fibroblasts (MEFs, Fig. 1e). *WT*, *Trp53*^*wm-R172H/+*^ and *Trp53*^*wm-R245W/+*^ mice also showed no significant differences in survival (Fig. 1f). *Trp53*^*wm-R172H/−*^ and *Trp53*^*+/−*^ mice were tumor-prone as expected and exhibited similar survival as well (Fig. 1g). Thus, detailed characterization of the *Trp53*^*wm-R172H*^ and *Trp53*^*wm-R245W*^ alleles showed they express WT p53 that maintains normal function.

### Somatic *Trp53* mutations differentially drive mammary tumors

**Fig. 2:**
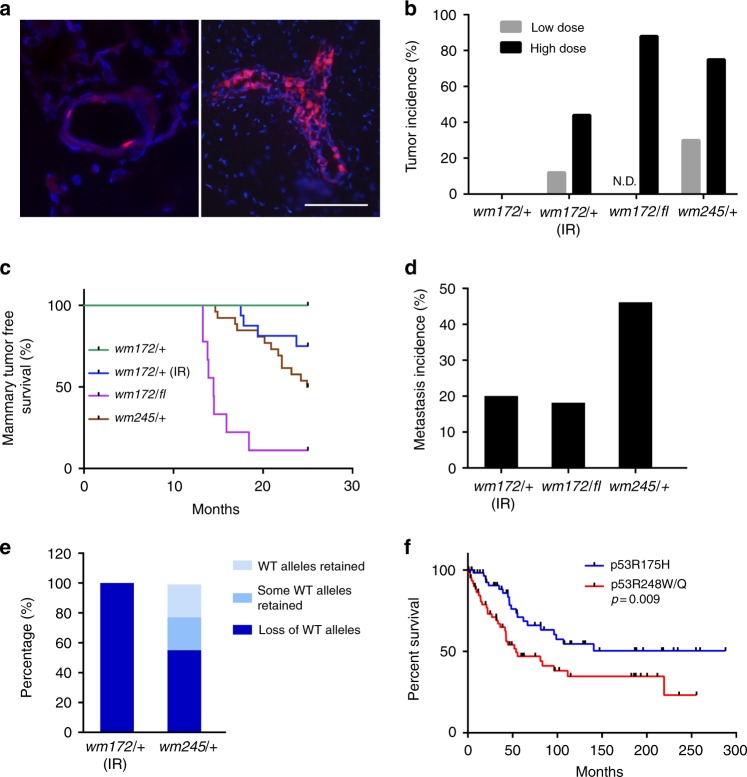
Somatic *Trp53R172H* and *Trp53R245W* mutations in mouse mammary epithelium drive tumorigenesis. **a** Representative pictures of mouse mammary glands from *Rosa26-LSL-tdTomato* mice after Ad-Cre injection in mammary ducts. Two doses of Ad-Cre were deployed for mammary intraductal injection, with low dose infecting 1–5% epithelial cells (left), and high dose 50–70% (right). Scale bar shows 100 μm and applies to both sections. **b** Comparison of the tumor incidences from mice of various *Trp53* genotypes (*wm172/+*, *Trp53wm-R172H/+*; *wm172/fl*, *Trp53wm-R172H/fl*; *wm245/+*, *Trp53wm-R245W/+*) after low vs. high doses of Ad-Cre injection. IR, γ-radiation. For animal numbers in each group, please refer to Table 1. N.D., non-determined. **c** Kaplan–Meier mammary tumor free survival curves of Ad-Cre injected mice with various *Trp53* genotypes (*wm172/+*, *wm-R172H/+*, *n* = 21; *wm172/+* (IR), *wm-R172H/+* (γ-radiation), *n* = 17; *wm172/fl*, *wm-R172H/fl*, *n* = 10; *wm245/+*, *wm-R245W/+*, *n* = 25). **d** Comparison of metastasis incidences of mammary tumors from Ad-Cre-injected mice with various *Trp53* genotypes (*wm172/+* (IR), *wm-R172H/+* (γ-radiation), *n* = 5; *wm172/fl*, *wm-R172H/fl*, *n* = 11; *wm245/+*, *wm-R245W/+*, *n* = 13). **e** The status of the WT *Trp53* allele in mammary tumors from Ad-Cre-injected mice with various *Trp53* genotypes (*wm172/+* (IR), *wm-R172H/+* (γ-radiation), *n* = 5; *wm245/+*, *wm-R245W/+*, *n* = 9). **f** Kaplan–Meier survival curves of human breast, ovarian and lung cancer patients containing *TP53R175H* (*n* = 62) or *TP53R248W/Q* (*TP53R248W* or *TP53R248Q*, *n* = 65) mutations. *p* = 0.009 (*t*-test)

To generate a somatic breast cancer model, we injected adenoviruses expressing Cre recombinase (Ad-Cre) into the mammary ducts of *Trp53*^*wm-R172H/+*^ and *Trp53*^*wm-R245W/+*^ mice. Modulating the dose of Ad-Cre allowed us to induce recombination of the *Rosa26-LSL-tdTomato* (a conditional allele that expresses tomato red fluorescent protein in response to Cre^13^), and likely *Trp53* in 1–5% (low dose) or 50–70% (high dose) of epithelial cells (Fig. 2a). None of the 21 injected *Trp53*^*wm-R172H/+*^ mice developed mammary tumors by 25 months post Ad-Cre injection (Fig. 2b, c and Table 1). To enhance tumor development, Ad-Cre-injected *Trp53*^*wm-R172H/+*^ female mice were exposed to γ-radiation. One of the eight mice (low dose Ad-Cre) developed a mammary tumor at 23.7 months, and four of nine mice (high dose Ad-Cre) at 18.3 months on average (Fig. 2b, c and Table 1). Only one tumor metastasized to lung (Fig. 2d). All *Trp53*^*wm-R172H/+*^ females treated with Ad-Cre and γ-radiation showed loss of the WT *Trp53* allele (Fig. 2e). To examine the potential cooperative role of loss of heterozygosity (LOH), cohorts of *Trp53*^*wm-R172H/fl*^ female mice were established, in which the conditional *Trp53*^*fl*^ allele is simultaneously deleted when *Trp53*^*wm-R172H*^ becomes mutant in response to Cre. Remarkably, following high dose Ad-Cre injection, the *Trp53*^*wm-R172H/fl*^ mice developed mammary tumors with a high frequency (90%), at around 14.7 months on average (Fig. 2b, c and Table 1). Two of 11 tumors disseminated to lung (Fig. 2d). These data suggest a strong inhibitory role of WT p53 against p53R172H-induced mammary tumorigenesis.

**Table 1:** Summary of mammary tumors developed from the *Trp53*^*wm-R172H/+*^ (with or without γ-radiation treatment), *Trp53*^*wm-R172H/fl*^ and *Trp53*^*wm-R245W/+*^ mice subjected to low- or high-dose Ad-Cre injection

Mouse genotype	Treatment	Virus dose (mouse numbers)	Tumor (metastasis)	Latency^a^ (months)
*p53* ^*wm-R172H/+*^	Ad	High (2)	0 (0)	N/A
	Ad-Cre	Low (10)	0 (0)	N/A
		High (11)	1^b^ (0)	25.4^b^
	Ad-Cre+IR	Low (8)	1 (0)	23.7
		High (9)	4 (1)	18.3
*p53* ^*wm-R172H/fl*^	Ad-Cre	High (10)	11 (2)	14.7
*p53* ^*wm-R245W/+*^	Ad	High (2)	0 (0)	N/A
	Ad-Cre	Low (13)	4 (1)	23.2
		High (12)	9 (5)	18.8

^a^Referring to time after Ad or Ad-Cre injection

^b^Mice were generally euthanized around 25 months after Ad-Cre injection if no tumors were formed, except for this female, which was kept for longer time accidently and exhibited a tumor at 25.4 months post Ad-Cre injection.

IR, γ-radiation

In contrast, *Trp53R245W* heterozygosity was sufficient for mammary tumorigenesis. Thirteen of 25 *Trp53*^*wm-R245W/+*^ mice injected with Ad-Cre at either dose developed tumors (Fig. 2b, c and Table 1) with 46% metastasis to lung and liver (Fig. 2d). p53R245W tumors showed more variation in LOH with 55.6% tumors having LOH, 22.2% tumors without LOH, and 22.2% retaining WT alleles in some cells (Fig. 2e and Supplementary Fig. 3). The efficiency of Cre-mediated recombination was similar for both alleles (Supplementary Fig. 4).

To increase the power to identify differences between missense mutations at *TP53R175* and *TP53R248* in human patients, we analyzed a combined dataset of patients who developed one of three epithelial cancers (breast, ovarian, and lung carcinomas), and found that individuals with *TP53R248* mutations (tryptophan or glutamine) exhibited a significant worse survival compared to ones with the *TP53R175H* mutation (Fig. 2f).

**Fig. 3:**
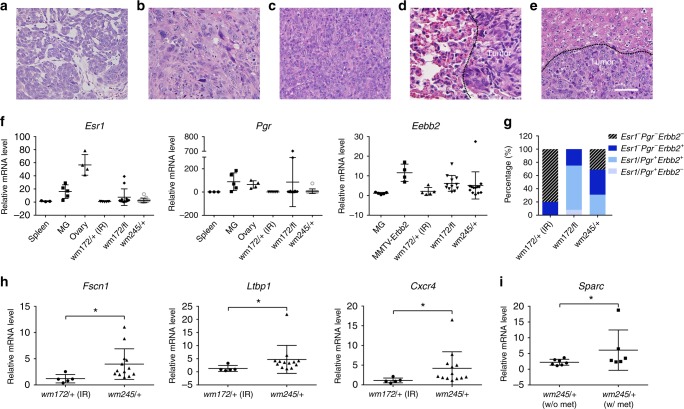
Characteristics of the p53R172H and p53R245W mammary tumors **a**–**e** Hematox;ylin and Eosin-stained sections of mammary tumors from mice showing different pathological subtypes. **a** A moderately-differentiated adenocarcinoma. Note glandular and nesting formation of tumor composition; **b** A highly anaplastic cellular manifestation including spindle cell, cellular pleomorphism and loss of glandular and differentiation features; **c** A sarcomatoid carcinoma with an undifferentiated sarcoma-like manifestation. **d** A localized metastatic adenocarcinoma in lung. Tumor nodule displays glandular proliferation of well-to-moderately-differentiated adenocarcinoma of mammary origin; **e** A metastatic tumor to liver. The tumor displays the same phenotypic anaplasia seen in primary tumor (**b**). Note the distinct boundary of metastases and normal tissues, as marked by the dashed line. Scale bar is 50 μm and applies to all sections. **f** RT-qPCR analysis for *Esr1*, *Pgr* and *Erbb2* in WT mouse spleens, mammary glands (MG), or ovaries, or mammary tumors from the *MMTV-Erbb2* mice, and tumors from Ad-Cre-injected mice of various *Trp53* genotypes (*wm172/+*, *wm-R172H/+*; *wm172/fl*, *wm-R172H/fl*; *wm245/+*, *wm-R245W/+*). IR, γ-radiation. Error bars, s.d. **g** Summary of the molecular subtypes of mammary tumors from mice of various genotypes, based on data in f. *Esr1/Pgr*^*+*^ label means *Esr1* postive or *Pgr* positive, or both. **h** RT-qPCR analysis for *Fscn1*, *Ltbp1,* and *Cxcr4*, in Trp53R245W-driven mammary tumors compared to Trp53R172H mammary tumors. Error bars, s.d. * *p* < 0.05 (*t*-test) **i** RT-qPCR analysis for *Sparc* in Trp53R245W-driven mammary tumors that had metastasis compared to those without metastasis. Error bars, s.d. * *p* < 0.05 (*t*-test)

Pathological examination identified all tumors from *Trp53*^*wm-R172H/+*^ (irradiated) and *Trp53*^*wm-R172H/fl*^ mice as adenocarcinomas (Supplementary Fig. 5). In contrast, tumors from the *Trp53*^*wm-R245W/+*^ mice were more diverse: adenocarcinoma (76.9%), anaplastic carcinoma (15.4%) and sarcomatoid carcinoma (7.7%) (Supplementary Fig. 5), with distinct morphologies (Fig. 3a–c). Metastases exhibited morphological similarities with the associated primary tumors (Compare Fig. 3d to Fig. 3a and Fig. 3e to Fig. 3b). To identify the molecular subtypes of tumors, we measured expression of *Esr1*, *Pgr*, and *Erbb2*. All five mammary tumors from *Trp53*^*wm-R172H/+*^ mice (Ad-Cre plus IR) exhibited minimal level of the *Esr1* and *Pgr* transcripts, and only one of these tumors showed higher expression of *Erbb2*, making them predominantly triple negative (80%, Fig. 3f, g). In contrast, the p53R245W-driven tumors exhibited more diverse molecular subtypes: *Esr1*^*−*^*Pgr*^*−*^*Erbb2*^*−*^ (triple negative, 30.8%), *Esr*^*−*^*Pgr*^*−*^*Erbb2*^*+*^ (Her2 enriched, 38.4%), or *Esr1/Pgr*^*+*^*Erbb2*^*+*^ (*Esr1* or *Pgr*, or both, positive, most likely luminal B, 30.8%) (Fig. 3f, g). The above association between specific *Trp53* hot spot mutations and breast tumor subtype phenocopies human breast cancers, where p53R175 mutations were most frequently identified in triple negative breast cancer and p53R248 mutations in triple negative, Her2 enriched and luminal B breast cancers^2^. In the *Trp53*^*wm-R172H/fl*^ mice, loss of the WT allele altered the subtype spectrum of p53R172H driven tumors. The majority of tumors from these mice was *Esr1/Pgr*^*+*^*Erbb2*^*+*^ (66.7%), and the rest were *Erbb2* positive (25.0%) or *Esr1*^*−*^*Pgr*^*+*^*Erbb2*^*−*^ (8.3%) (Fig. 3f, g).

Previously, a panel of genes that mark or mediate human breast cancer metastasis to the lungs have been identified^14^. We therefore examined whether expression of 18 of these genes, which showed significance either from transcriptomic analysis or functional validation, was also enriched in the p53R245W mammary tumors, which had high metastatic incidence (Fig. 2d). Strikingly, seven genes were significantly highly expressed in p53R245W tumors as compared to the less metastatic p53R172H tumors (Fig. 3h and Supplementary Fig. 6). In addition, *Sparc*, a matricellular glycoprotein associated with human breast cancer aggressiveness^14,15^, was expressed significantly higher in metastatic versus non-metastatic p53R245W tumors (Fig. 3i). Thus, the data suggest that expression of *Trp53* missense mutations in a few mammary epithelial cells results in aggressive breast cancers that share many features of human breast cancers.

### Genomic heterogeneity in somatic p53R245W mammary tumors

**Fig. 4:**
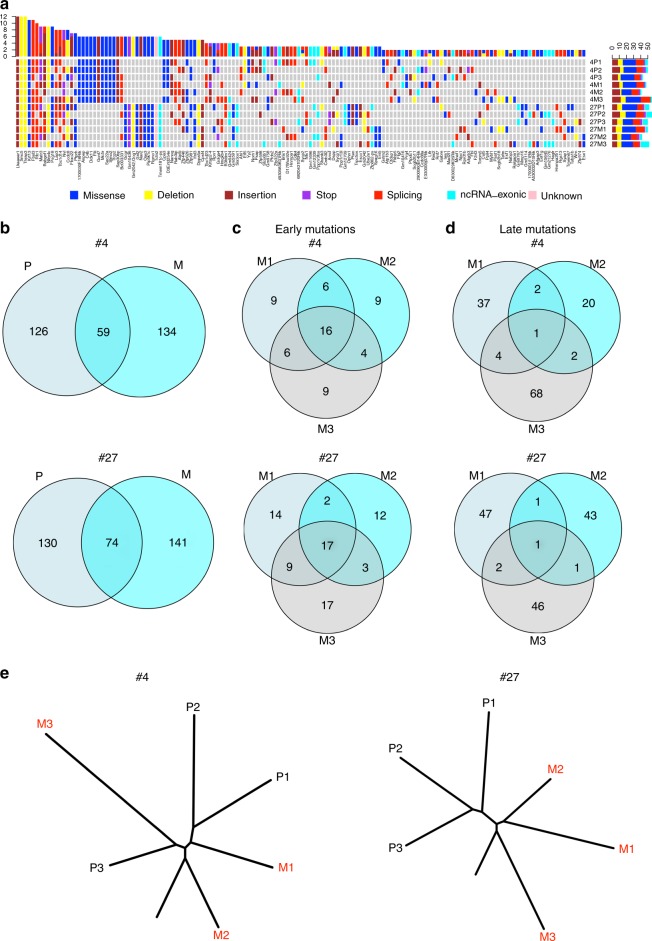
Genomic heterogeneity and mutational evolution in mammary tumors driven by somatic Trp53R245W mutant. **a** Heatmaps of genes with various alterations identified in at least two tumors or metastases of the three physically separated regions of primary tumors (P1–P3) and associated metastatic clones (M1–M3) from each of two *Trp53*^*wm-R245W/+*^ mice (#4 and #27), subjected to high dose Ad-Cre intraductal injection. Note that some genes have more than one alterations. **b** Venn diagrams depicting the overlap of the identified mutations in primary tumors (P) with those in the associated metastases (M) in mouse #4 and #27. **c** Venn diagrams depicting the overlap of the identified early mutations among all three metastatic clones (M1–M3) in mouse #4 and #27, respectively. **d** Venn diagrams depicting the overlap of the identified late mutations among all three metastatic clones (M1–M3) in mouse #4 and #27, respectively. **e** Phylogenetic trees generated by clustering genome-wide mutation data from 3 multi-region primary tumor samples (P1–P3) and 3 metastatic clones (M1–M3) for both mouse #4 and #27. Relative branch lengths were determined from the proportion of mutations in each branch

In recent years, genomic sequencing has allowed analysis of the genetic basis of tumor progression and metastasis in different types of cancers, including breast cancer^16^, which may enable tailoring of therapeutic strategies to the fundamental molecular lesions driving a particular tumor. The faithful recapitulation of many features of human breast cancer makes our p53R245W mammary tumor model a useful tool for understanding invasive breast tumors driven by p53R245W. We performed whole exome sequencing of three spatially separated regions from two primary mammary adenocarcinomas and associated lung metastasis (3 for each primary tumor) from *Trp53*^*wm-R245W/+*^ mice. All tumors and normal lung controls were sequenced at a depth averaging 96.4×. Mouse #4 had 185 mutations present in primary tumor site(s), and 193 mutations in metastatic site(s). Mouse #27 had 204 mutations in primary site(s) and 215 in metastatic site(s). The majority of these were missense and splice site mutations (Fig. 4a and Supplementary Data 1). Interestingly, genes that are involved in chromosome replication, segregation, or repair, such as *Smc5*^17^ and *Rfc1*^18^, or well-known tumor suppressors, such as *Pten*^19^, were among the list. It is also noteworthy that in addition to the *TrP53* mutation, there were 15 other mutations that were shared among all sequenced samples (Fig. 4a and Supplementary Data 1) for each mouse. These mutations may play critical cooperative roles in p53R245W-initiated tumorigenesis and further validation is needed.

Analysis of intra-tumor heterogeneity of samples revealed 19 and 23 mutations shared among three regions sampled in primary tumors for mice #4 and #27, respectively, equal to 10.3% and 11.3% of total mutations identified (Supplementary Fig. 7a). Plus, 8.8% (17/193) and 8.4% (18/215) of mutations were shared among three metastatic sites, respectively (Supplementary Fig. 7b). Among 319 mutations identified for both primary tumor and metastases in mouse #4, only 59 (18.5%) were shared between any primary tumor site and any metastatic site (Fig. 4b). Similarly, for mouse #27, 21.4% (74/345) mutations were shared between any primary tumor site and any metastatic site (Fig. 4b). These shared mutations are defined as early mutations since they most likely originated in primary sites. In addition, we identified 134 and 141 mutations for #4 and #27, respectively, which were exclusively present in metastases and therefore were acquired after metastatic cells left the primary site and designated as late mutations. 27.1% (16/59) and 23.0% (17/74) early mutations were shared among three metastatic sites in #4 and #27 mice, respectively (Fig. 4c). In striking contrast, there was only 0.75% (1/134) shared late mutations in mouse #4 and 0.71% in mouse #27 (1/141) (Fig. 4d). These results suggest tumor cells with metastatic potency preexist in the parental tumors.

Inter-patient heterogeneities have long been observed in different human cancer types^16^. Among the mutations identified in primary tumors from mouse #4 and #27, 13.4% were shared between the two (Supplementary Fig. 7c, Left). In addition, 9.97% were shared between metastatic tumors from these mice (Supplementary Fig. 7c, Right).

### Mutation evolution in somatic R245W mammary tumors

Multi-region exome sequencing also allows delineating the clonal and sub-clonal evolutionary architecture of primary tumors and metastasis^20,21^. All specimens from primary and metastatic lesions showed consistent truncal mutations of similar length suggesting that both tumors shared a similar timing of subclonal progression (Fig. 4e). Branching revealed two different evolving patterns of metastatic progression from primary lesions. Two metastatic sites, M1 and M2, in mouse #4 diverged away from the primary tumor early even before the phylogenetic diversification of the primary tumor. However, the third metastatic site, M3, did not emerge until the primary tumor diversified. M3 shared the highest percentage of mutations with the primary subclone P3 (Fig. 4e, left), suggesting P3 was the origin of this metastatic site. Thus, the mammary tumor from mouse #4 exhibits a multi-origin metastatic pattern. In mouse #27, all three metastatic sites, M1–M3, diverged away from the primary tumor early before the primary tumor diversified phylogenetically, with M2 requiring the fewest number of mutations to depart and M3 the highest (Fig. 4e, right).

One might argue that the above multi-origin of metastases is due to independent seeding coming from individual primary tumors that interdigitate together. However, the fact that 15 truncal mutations were shared among all samples for both mice (Fig. 4a and Supplementary Data 1) argues against two entirely independent lineages generated from different initiating cells. The primary and metastases shared similar allelic frequencies for the majority of the truncal mutations (Supplementary Fig. 8), further supporting the scenario where only one primary clone exists.

The above findings are clinically relevant and indicate that treating actionable mutations that are subclonal in the primary tumor may not prevent disease relapse. The extremely early dissemination observed in mammary tumors from mouse #4 and #27 also warrants the necessity of closely monitoring breast cancer patients containing the *TP53R248W* mutation even when the primary lesion is discovered and treated at a very early stage.

## Discussion

The strikingly high frequency of *TP53* mutation in human cancers and early studies showing the transformation ability of mutant TP53 in cultured cell lines make it unquestionably critical to examine outcomes in genetically modified mice. In our current study, we generated two conditional *Trp53*^*wm-R172H*^ and *Trp53*^*wm-R245W*^ alleles, which allow mutant p53 expression upon Cre-mediated recombination. Importantly, in the absence of Cre, the *Trp53*^*wm-R172H*^ and *Trp53*^*wm-R245W*^ alleles express functional WT p53, leading to several advantages over all other conditional mutant *Trp53* alleles. 1) the *Trp53*^*wm/+*^ mice express normal levels of WT p53 and are not tumor prone; 2) mutant p53 is converted from WT at the *endogenous* locus closely mimicking the clinical situation; 3) mutant p53 is induced in the specific tissue/cell of interest, limiting tumor development to a single tissue; 4) the tumor microenvironment and immune system of the animals are p53 WT.

The mammary tumors that developed from our models recapitulated a number of characteristics of human breast cancers. Tumors from the *Trp53*^*wm-R172H/+*^ mice were largely triple negative, whereas those from the *Trp53*^*wm-R245W/+*^ mice were triple negative, Her2 positive, and luminal B, faithfully phenocoping the association of different *TP53* hot spot mutations with specific breast cancer subtypes in humans^2^. In addition, the *Trp53R245W* mutation led to highly metastatic mammary tumors correlating to shortened survival in patients with epithelial cancers that contain *TP53R248* mutations. Moreover, some, although not all, genes identified previously to mark human breast cancer metastasis to lung were also highly expressed in the p53R245W driven mammary tumors that had higher lung metastasis incidence compared to p53R172H tumors.

Emerging in vivo evidence shows that hot spot *p53* mutations are not functionally equal. For example, germline *Trp53R172H* and *Trp53R270H* mice exhibited significant differences in their tumor spectra^22^. In addition, in human *TP53* Knock-in (HUPKI) models expressing the p53R248Q and p53G245S mutants, *TP53*^*R248Q/−*^ mice showed decreased survival, faster tumor development, and a broader tumor spectrum when compared to *TP53*^*G245S/−*^ animals^23^. In our study, somatic p53R172H was less potent in initiating mammary tumorigenesis compared to p53R245W, and the p53R245W tumors had a higher frequency of metastasis. It will also be interesting to investigate whether and how these p53 mutants differentially impact tumor cells in drug responses/resistance. It is not clear why these hot spot *Trp53* mutations exhibit different magnitudes of effects. However, it is noteworthy that the above-mentioned differences indicate that p53 structural mutants (mouse p53R172H and human p53G245S) are less potent than contact mutants (mouse p53R245W, and human p53R248Q). We speculate that, due to their different protein structures, these two categories of p53 mutants may have a distinct binding partners, which may differentially modify their impact. Previously, it has been shown that p53 mutants regulate gene expression through interacting with transcriptional factors^24^. A comparison of the transcriptional targets of these different p53 mutants in our breast tumors with chromatin immunoprecipitation combined with sequencing, and/or 3’-RNA sequencing is needed. Furthermore, in contrast to p53 contact mutants, the p53 structural mutants, due to their drastic structure alterations, may have a weaker interaction with the WT p53 and therefore lead to a dampened dominant-negative effect. This speculation is supported by our observation that all the p53R172H mammary tumors examined underwent LOH, while the WT *Trp53* allele was retained in some p53R245W tumors.

The evident metastasis frequency of the mammary tumors driven by *Trp53R245W* mutation also allowed us to expand our understanding of the metastatic paradigm. So far, two patterns have been proposed for metastasis evolution^25^. Parallel evolution, where seeding from an ancestral clone occurs early during disease progression, resulting in a branched pattern of evolution. In contrast, when spreading occurs late in the evolution of the primary tumor, a linear pattern of evolution is observed. In our study, two p53R245W tumors displayed parallel evolution with a main trunk and distinct splits in both cases. Remarkably, in mouse #27, all the metastatic clones diverge away from the primary tumor *before* the subclonal diversification of the primary lesion occurs, indicating extremely early dissemination. This finding has important clinical implications. In contrast to tumors with linear evolution, patients with tumors that evolve in a branched fashion, e.g., TP53R248W breast tumors, may require closer monitoring even when the primary lesion is discovered and treated at a very early stage, due to the early spreading of tumor cells.

It is not clear why different evolution patterns exist among different tumors. One can speculate that it may reflect the intrinsic property of the initial oncogenic driver(s). Strong oncogenic driver(s), e.g., Trp53R245W in our study, after inducing a handful of mutations and/or genomic instability, may have endowed tumor cells sufficient metastatic capability so that further mutations are not required for those cells to depart the primary site. In fact, early systemic spread has also been observed in mouse breast tumors driven by *Her2* or *polyomavirus-middle T*, two well-known aggressive oncogenes^26^. In contrast, in the linear evolution model, tumor cells in the primary disease initiated by relatively weak driver(s) probably demand a large repertoire of mutations, which takes considerable time to accumulate, before they gain the ability to spread.

These invaluable models highlight the aggressive nature of *p53* somatic mutations and the parallel tumor evolution pattern driven by a *p53* missense mutation as the initiating event. In addition to studying breast cancers, the *Trp53*^*wm-R172H*^ and *Trp53*^*wm-R245W*^ alleles can also be used to investigate the role of p53 mutations in driving other cancers. Additionally, they can be used to determine how p53 mutant cells modify and shape their microenvironment. Mouse models will provide a better understanding of the mechanisms underlying tumor initiation and progression, which will lead to more rational drug development and testing.

## Methods

### Generation of *Trp53*^*wm-R172H*^ and *Trp53*^*wm-R245W*^ mouse alleles

The targeting vectors were generated by cloning a cDNA fragment that contained exons 5–11 (for the *Trp53*^*wm-R172H*^ allele) or 7–11 (for the *Trp53*^*wm-R245W*^ allele), as well as a polyadenylation signal, followed by an *Frt*-flanked *PGKpuro* cassette into intron 4 (for the *Trp53*^*wm-R172H*^ allele) or an *Frt*-flanked *PGKneo* cassette into intron 6 (for the *Trp53*^*wm-R245W*^ allele) of a *Trp53* genomic fragment. The cDNA fragment and *PGKpuro* or *PGKneo* cassette was flanked by *loxp* sequences. An arginine-to-histidine substitution (CGC to CAC) at codon 172 or arginine-to-tryptophan substitution (CGC to TGG) at codon 245 was performed by site-directed mutagenesis for the *Trp53*^*wm-R172H*^ and *Trp53*^*wm-R245W*^ alleles, respectively (details in Supplementary Fig. 1a). The resulting constructs were then individually cloned into a vector containing a thymidine kinase (TK) cassette. The entire targeting vectors were sequence verified, linearized with NotI, and electroporated into AB-1 embryonic stem (ES) cells. DNA from G418-resistant FIAU-sensitive ES colonies was subjected to Southern blot analysis (Supplementary Fig. 1c, d and Supplementary Fig. 9). For each allele, two targeted ES cell clones were injected into C57BL/6 blastocysts and transferred into pseudo pregnant CD1 female recipients. The resulting chimeras were mated with C57BL/6 females to secure germline transmission of the mutant alleles. Southern blot analysis was performed to reassure correct targeting in F1 mice, or a combination of primers internal and external to the targeting vectors was used to amplify the alleles and PCR products were sequenced to ensure absence of other alternations. Mice with the targeted alleles were crossed with *Rosa26-flp* mice (also in the C57BL/6 background) to remove the *PGKpuro* or *PGKneo* cassettes. The resulting mice were maintained in a mixed C57BL/6/129 S strain background. All mouse experiments were carried out in compliance with the guidelines of the American Association for Accreditation for Laboratory Animal Care International and the US Public Health Service Policy on Humane Care and Use of Laboratory Animals. Study protocols were approved by M.D. Anderson Cancer Center Institutional Animal Care and Use Committee.

### Genotyping

Genotyping was performed by polymerase chain reaction (PCR) using primers surrounding the 5′ *loxp* site for both alleles. The primers used to genotype both alleles were as follows: *Trp53*^*wm-R172H*^ forward, 5′-AGCTGCTAGAGACAGTTGAGG-3′, reverse, 5′-GCCAGCAAAGAGAGGACT-3′; *Trp53*^*wm-R245W*^ forward, 5′-ACCTTATGAGCCACCCGA-3′, reverse: 5′-GGAAGACACAGGATCCAGGT-3′.

### Cell culture, apoptosis and EDU assays

MEFs were generated by crossing the appropriate mice and collecting embryos at 13.5 dpc. MEFs were maintained in Dulbeco’s modified Eagle’s medium (DMEM) supplemented with 10% fetal bovine serum (FBS) and penicillin (100 IU/ml)/streptomycin (100 mg/ml). Apoptosis was measured in freshly isolated thymocytes from irradiated mice 4 h after 6 Gy radiation, using the annexin-V assay (Clontech) with fluorescence-activated cell sorting (FACS) analysis. EdU labeling and flow cytometry were performed following the manufacturer’s specifications (ThermoFisher Scientific). Briefly, MEFs of various genotypes, 4 h after 6 Gy γ-radiation, were labeled with EdU followed by fixation. Incorporated Edu was quantified by FACS analysis.

### Western blot assay and reverse transcription PCR

Tissues were homogenized and protein lysates were prepared for western blots. The antibodies used were: p53, 1:1000 (NCL-L-p53-CM5p, Leica^27^), β-actin, 1:10000 (A5441, Sigma-Aldrich^28^) and Vinculin, 1:5000 (V9131, Sigma-Aldrich^29^). For full Western blot images, please refer to Supplementary Fig. 10. Total RNA was extracted from the tissues using TRIzol reagent (Life Technologies). The first-strand cDNA synthesis kit (GE Healthcare) was used for reverse transcriptase reactions. Reverse transcription quantitative PCR (RT-qPCR) was performed according to the manufacturer’s specifications (Life Technologies). Data were normalized to *β-actin* or *Rplp0*. Primers used for RT-qPCR of *Rplp0*, *Cdkn1a* (*p21*) and *Bbc3* (*Puma*) were as follows: *Rplp0* forward, 5′-CCCTGAAGTGCTCGACATCA-3′, reverse, 5′-TGCGGACACCCTCCAGAA-3′; *Cdkn1a* forward, 5′-CCTGACAGATTTCTATCACTCCA-3′, reverse, 5′-CAGGCAGCGTATATCAGGAG-3′; *Bbc3* forward, 5′-GCGGCGGAGACAAGAAGA-3′, reverse 5′-AGTCCCATGAAGAGATTGTACATGAC-3′;^30,31^. Primers used for RT-qPCR of other targets were as follows: *Fscn1* forward, 5′-CAGCCAATCAGGATGAAGAG-3′, reverse, 5′-CACCACTCGATGTCAAAGTAG-3′; *Ltbp1* forward, 5′-GAGAGTCCAGGAAGGATACA-3′, reverse, 5′-GCTTGTCGGATGGTATGTAG-3′; *Cxcr4* forward, 5′-TAACCACCACGGCTGTA-3′, reverse, 5′-CGACTATGCCAGTCAAGAAG-3′; *Sparc* forward, 5′-ACTCTTCCTGCCACTTCT-3′, reverse, 5′-GTTGTTGCCCTCATCTCTC-3′; *Man1a1*, forward, 5′-ATGCACTTGTCCCACTTATC-3′, reverse, 5′-GCTTCGAGATCTGTCTTGTC-3′; *Nedd9* forward, 5′-TACGAGTACCCATCCAGATAC-3′, reverse, 5′-GATGGTGGAATGGCATAGAC-3′; *Mmp2* forward, 5′-AGGACTATGACCGGGATAAG-3′, reverse, 5′-TCCGGTCATCATCGTAGTT-3′.

### Mouse mammary gland intraductal injection

Mice were crossed to BALB/c strain to obtain *Trp53*^*wm-R172H/+*^ and *Trp53*^*wm-R245W/+*^ female mice that were 50% BALB/c and 50% mixed C57BL/6/129 S strain backgrounds. Mice of 9–12 weeks old were anaesthetized with Ketamine and Xylazine (10 μg and 1 μg per gram of body weight, respectively). Adenoviri expressing Cre (Baylor College of Medicine) were diluted in injection medium (phosphate-buffered saline supplemented with 0.1% trypan blue) and introduced into mammary ducts of the #4 inguinal glands (10 μl/gland), using a 50 μl syringe with a 30 gauge needle (Hamilton). Two doses of Ad-Cre were deployed, 5 × 10^4^ particles per μl and 5 × 10^6^ particles per μl, to infect around 1–5% or 50–70% mammary epithelial cells, respectively. All mice were injected with the same batch adenoviri to avoid batch-to-batch variation.

### LOH

LOH was examined by polymerase chain reaction (PCR) analysis using primers surrounding the mutated site in exon 5 of the *Trp53*^*wm-R172H*^ and exon 7 of the *Trp53*^*wm-R245W*^ alleles, respectively, followed by sequencing. The primers used to measure LOH in the *Trp53*^*wm-R172H/+*^ mice were previously described:^30^ forward: 5′-TACTCTCCTCCCCTCAATAAGCTATTC-3′ and reverse: 5′-AGTCCTAACCCCACAGGCGGTGTT-3′; and for *Trp53*^*wm-R245W/+*^ mice were: forward, 5′-CGGTTCCCTCCCATGCTA-3′, reverse: 5′-AGCGTTGGGCATGTGGTA-3’. The criteria for determining the status of WT *Trp53* alleles are described in Supplementary Fig. 3.

### Histology

Tissues were fixed in 10% v/v formalin and embedded in paraffin. Sections were stained with haematoxylin and eosin (H&E).

### Patient survival analysis

The patient survival data were analyzed by the “Survival” R package that was downloaded from https://cran.rstudio.com/bin/macosx/mavericks/contrib/3.3/survival_2.41-3.tgz. The survival data were retrieved from the cBioportal for Cancer Genomics website. Tumor sample IDs were extracted based on the somatic mutations R175H, R248Q, and R248W for *TP53* gene. The clinical survival time and patient survival status were further retrieved from the cBioportal database based on the patient IDs. Five cancer datasets from cBioportal were used for the survival analysis: Pan-Lung Cancer (TCGA)^32^, Breast Cancer (METABRIC)^33,34^, Breast Invasive Carcinoma (TCGA, Provisional)^35^, Ovarian Serous Cystadenocarcinoma (TCGA, Provisional) and Ovarian Serous Cystadenocarcinoma (TCGA)^35^.

### Whole exome sequencing and the mutation calling

DNA was extracted from mouse samples at multiple tumor regions and submitted for the exome-seq sequencing at average 96.4 × coverage with Illumina Hi-seq 3000. The raw sequencing reads were mapped to mouse genome reference (GRCm38/mm10) with BWA alignment tool^36^. The aligned data were further subject to the processing steps as following: sorting, duplicates marking, base recalibration, generation of panel of normal and mutation calling. The analytical tools GATK and Mutect2 developed by the Broad Institute^37^ were utilized. The annotation of the somatic mutations was performed with ANNOVAR tool^38^. The mouse snp138 database and exome sequencing of three normal mouse lungs were used to filter the mouse single nucleotide polymorphisms.

### The phylogenetic analysis

The somatic mutations from primary and metastatic specimens were analyzed with the bioinformatics tool PHYLIP (Phylogeny Inference Package). The discrete character parsimony program (PARS) in PHYLIP package generated a phylogenetic tree. The tree was visualized by the drawtree function in PHYLP package.

### Statistical analysis

The number of biological replicates used for comparison is indicated in each figure. Student's *t-test* was performed with Prism 6 (GraphPad Software, San Diego, CA, USA). Differences were considered significant at a value of *p* < 0.05.

## Electronic supplementary material

Supplementary Information

Description of Additional Supplementary Files

Supplementary Data 1

## Data Availability

Raw FASTQ sequences have been deposited to NCBI Sequence Read Archive (SRA). The accession code is SRP155491. The data that support the findings of this study are available from the corresponding authors upon reasonable request.
