# Supplementary material for: Somatic Trp53 mutations differentially drive breast cancer and evolution of metastases
Source: Nat Commun. 2018 Sep 27;9:3953. doi: 10.1038/s41467-018-06146-9 (PMC6160420; doi:10.1038/s41467-018-06146-9)
Supplement: Supplementary file 1 — Supplementary Information [file 41467_2018_6146_MOESM1_ESM.pdf]

Somatic *Trp53* mutations differentially drive breast cancer and evolution of metastases

Zhang and Xiong et al.

Supplementary Figure 1

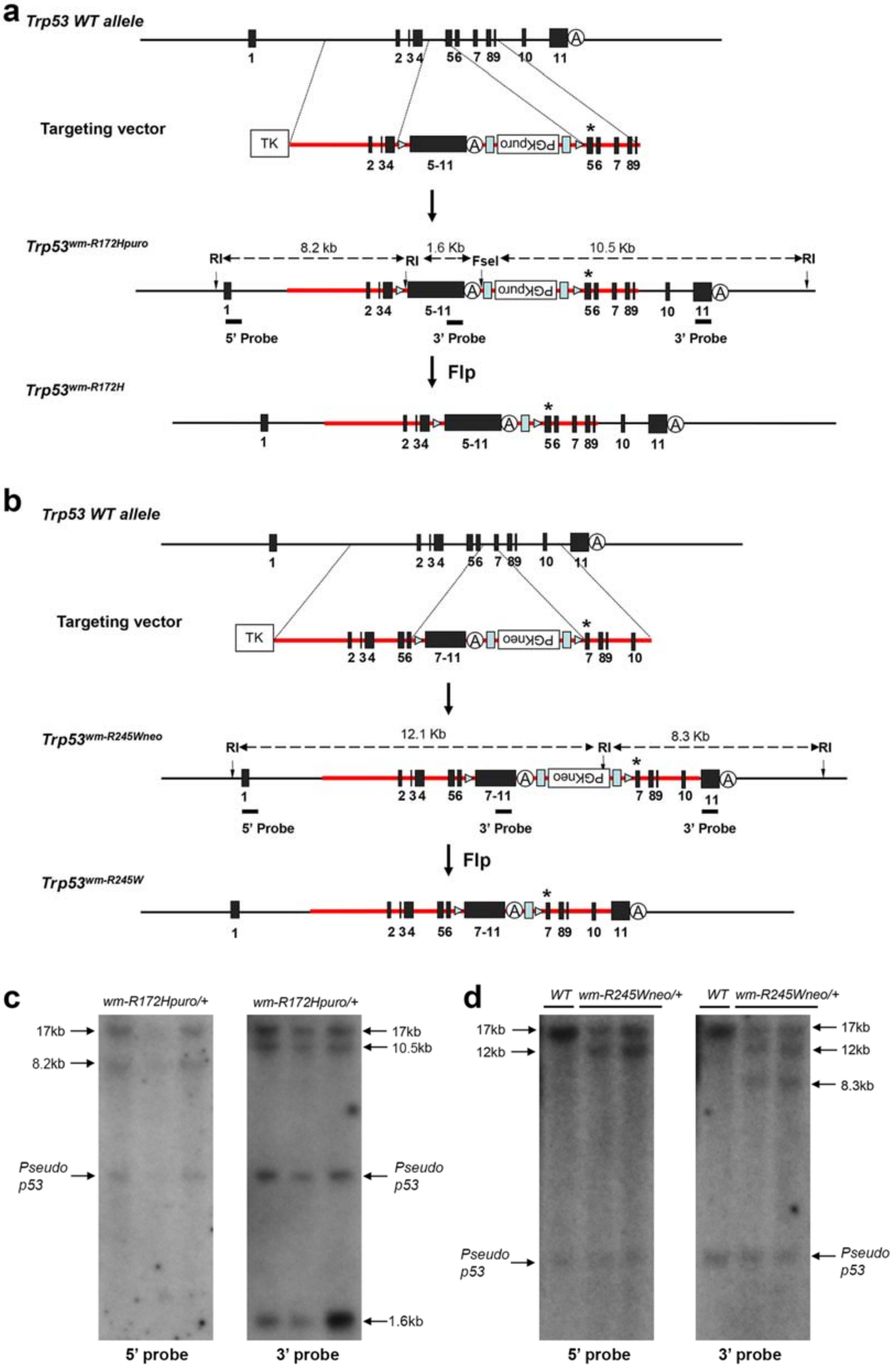

Supplementary Figure 1 Knockin strategy and confirmation of the proper targeting of the endogenous *Trp53* alleles

a. The *flp*-*Frt*-mediated strategy was used to generate the knockin *Trp53*$^{wm\text{-}R172H}$ allele. Two *loxPs* (blue triangles) flanking cDNA fragment for *Trp53* exon 5-11 and the *puromycin* (*puro*) resistance gene flanked by *Frt* sites (blue boxes) were inserted in intron 4 of mouse *Trp53* gene. In addition, 515G->C mutation (asterisk) was introduced in the following exon 5. The resulting mice were mated with CMV-*flp*-expressing mice to delete *puro*. The final product is the *Trp53*$^{wm\text{-}R172H}$ allele with a *loxP* flanked *Trp53* exon 5-11 cDNA and a single *Frt* site in intron 4, followed by a single substitution at nucleotide 515. Ⓐ*Trp53* native polyadenylation signaling sequence.

b. The *flp*-*Frt*-mediated strategy was used to generate the knockin *Trp53*$^{wm\text{-}R245W}$ allele. Two *loxPs* (blue triangles) flanked cDNA fragment for *Trp53* exon 7-11 and the *neomycin* (*neo*) resistance gene flanked by *Frt* sites (blue boxes) were inserted in intron 6 of mouse *Trp53* gene. In addition, 733C->T and 735C->G mutations (asterisks) were introduced in exon 7. The resulting mice were mated with CMV-*flp*-expressing mice to delete *neo*. The final product is the *Trp53*$^{wm\text{-}R245W}$ allele with a *loxP* flanked *Trp53* exon 7-11 cDNA and a single *Frt* site in intron 6, followed by substitutions at nucleotides 733 and 735. Ⓐ*Trp53* native polyadenylation signaling sequence.

c. Southern blot analysis was performed to confirm the proper targeting of the endogenous *Trp53*$^{wm\text{-}R172H}$ allele. The banding patterns for 5' and 3' probes after digesting the mouse genomic DNA with EcoRI (RI) and FseI are shown.

d. Southern blot analysis was performed to confirm the proper targeting of the endogenous *Trp53*$^{wm\text{-}R245W}$ allele. The banding patterns for 5' and 3' probes after digesting the mouse genomic DNA with EcoRI (RI) are shown.

Supplementary Figure 2

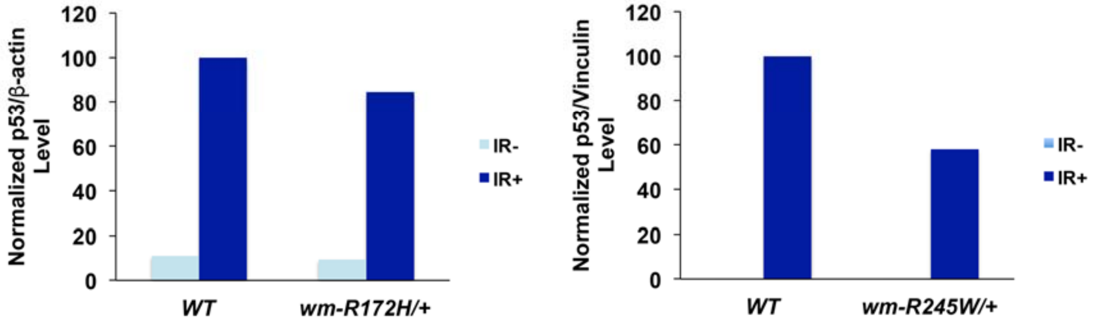

Supplementary Figure 2 Quantification of Western blot analysis for Trp53 protein levels in thymuses of various *Trp53* genotypes (*WT, wm-R172H/+ and wm-R245W/+*). IR, γ-radiation.

Supplementary Figure 3

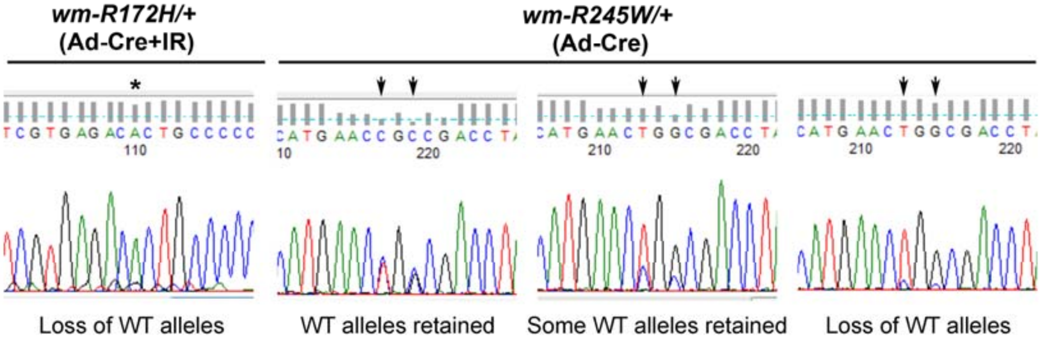

Supplementary Figure 3 Representative DNA chromatograms showing status of the *Trp53* WT allele in mammary tumors from Ad-Cre injected mice with different *Trp53* genotypes as shown. Loss of WT alleles was determined by a >80% reduction of the WT alleles, noted by the absence of the G nucleotide at position 515 (indicated by asterisk) for the $Trp53^{wm\text{-}R172H/+}$ mice and the C nucleotides at position of 733 and 735 (indicated by arrows) for the $Trp53^{wm\text{-}R245W/+}$ mice, respectively. "WT alleles retained" was determined by a <20% reduction of the WT alleles and "some WT alleles retained", 20-80% reduction of the WT alleles. IR, γ-radiation.

Supplementary Figure 4

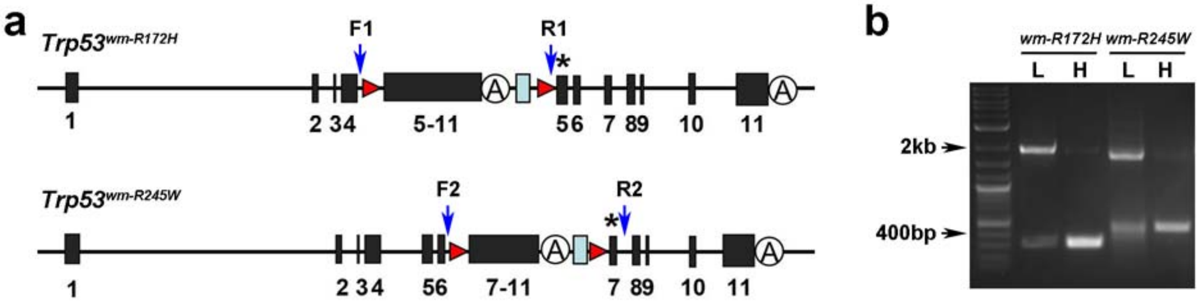

Supplementary Figure 4 Comparing the recombination of *Trp53$^{wm\text{-}R172H}$* and *Trp53$^{wm\text{-}R245W}$* alleles

a. Schematic representation of the *Trp53$^{wm\text{-}R172H}$* and *Trp53$^{wm\text{-}R245W}$* alleles. The priming positions of the pair of primers (F1/R1 and F2/R2) used for identifying allele recombination are indicated by blue arrows.

b. PCR with primers indicated in panel (a) produced DNA products for un-recombined (upper bands, close to 2kb) and recombined (lower bands, close to 400bp) *Trp53$^{wm\text{-}R172H}$* and *Trp53$^{wm\text{-}R245W}$* alleles. *wm-R172H*, mammary gland genomic DNA from *Trp53$^{wm\text{-}R172H/\ wm\text{-}R172H}$* mice subjected to intraductal injection of Ad-Cre; *wm-R245W*, mammary gland genomic DNA from *Trp53$^{wm\text{-}R245W/\ wm\text{-}R245W}$* mice subjected to intraductal injection of Ad-Cre. L, low dose of Ad-Cre; H, high dose of Ad-Cre.

Supplementary Figure 5

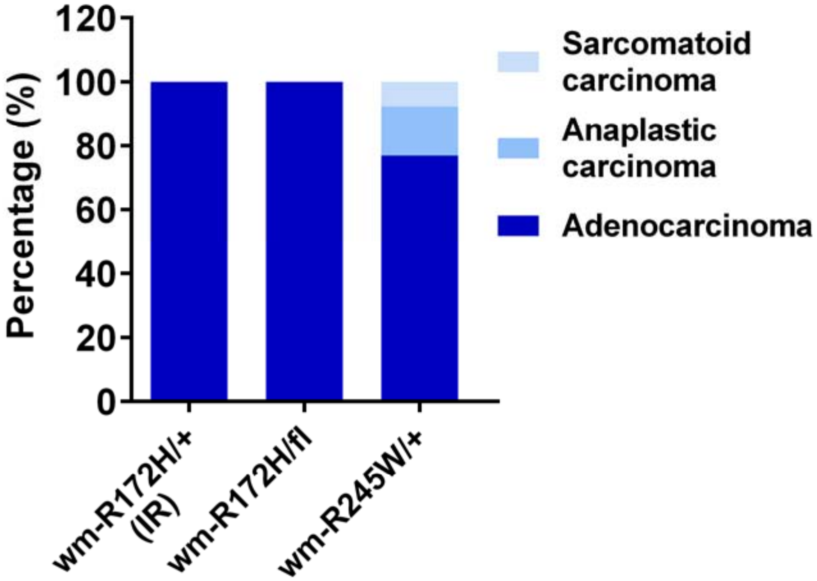

Supplementary Figure 5 Pathological subtypes of mammary tumors from Ad-Cre injected mice of various *Trp53* genotypes. IR, γ-radiation.

Supplementary Figure 6

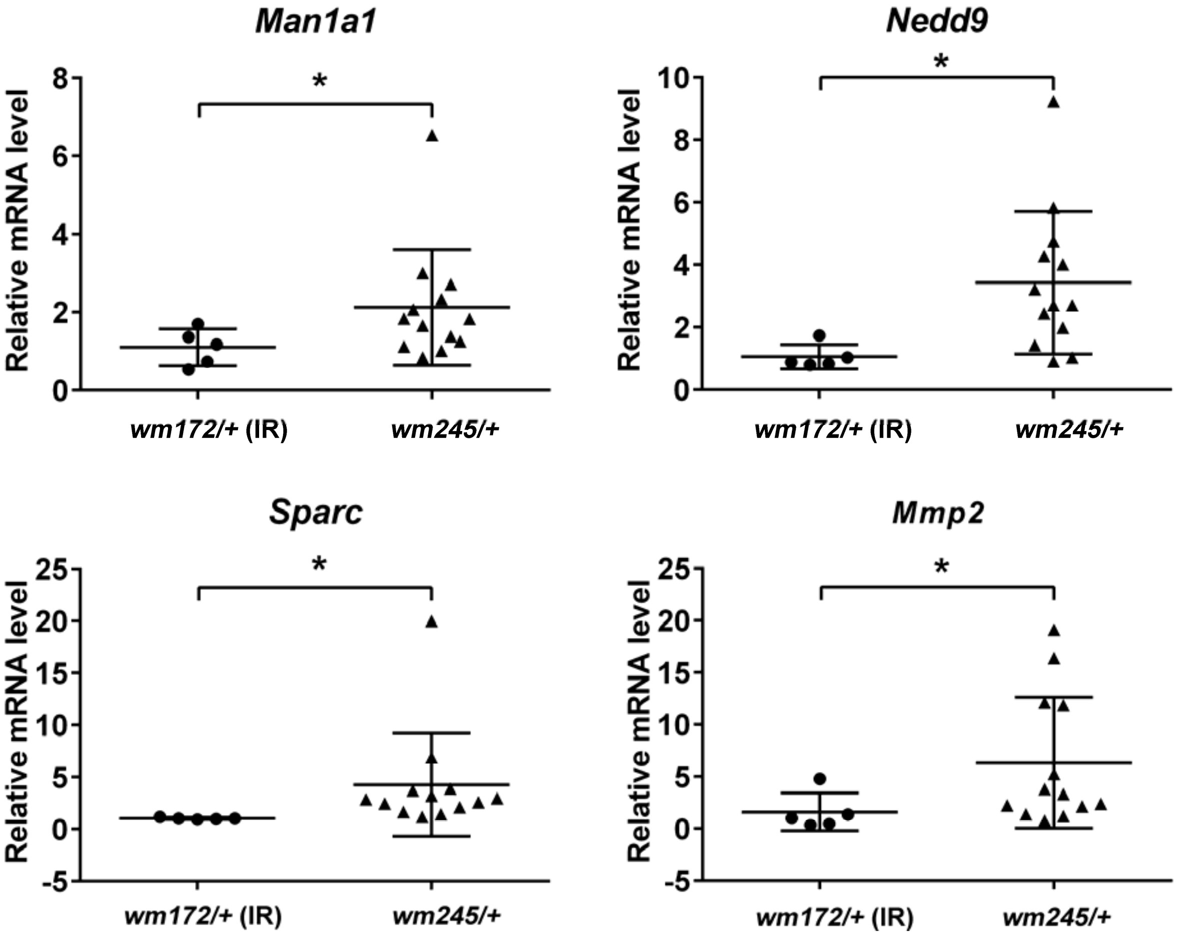

Supplementary Figure 6 RT-qPCR analysis for *Man1a1*, *Nedd9, Sparc* and *Mmp2*, in Trp53R245W driven mammary tumors compared to Trp53R172H mammary tumors. *wm172/+*, *Trp53*$^{wm\text{-}R172H/+}$; *wm245/+*, *Trp53*$^{wm\text{-}R245W/+}$; IR, γ-radiation. Error bars, s.d. * p < 0.05 (t-test)

Supplementary Figure 7

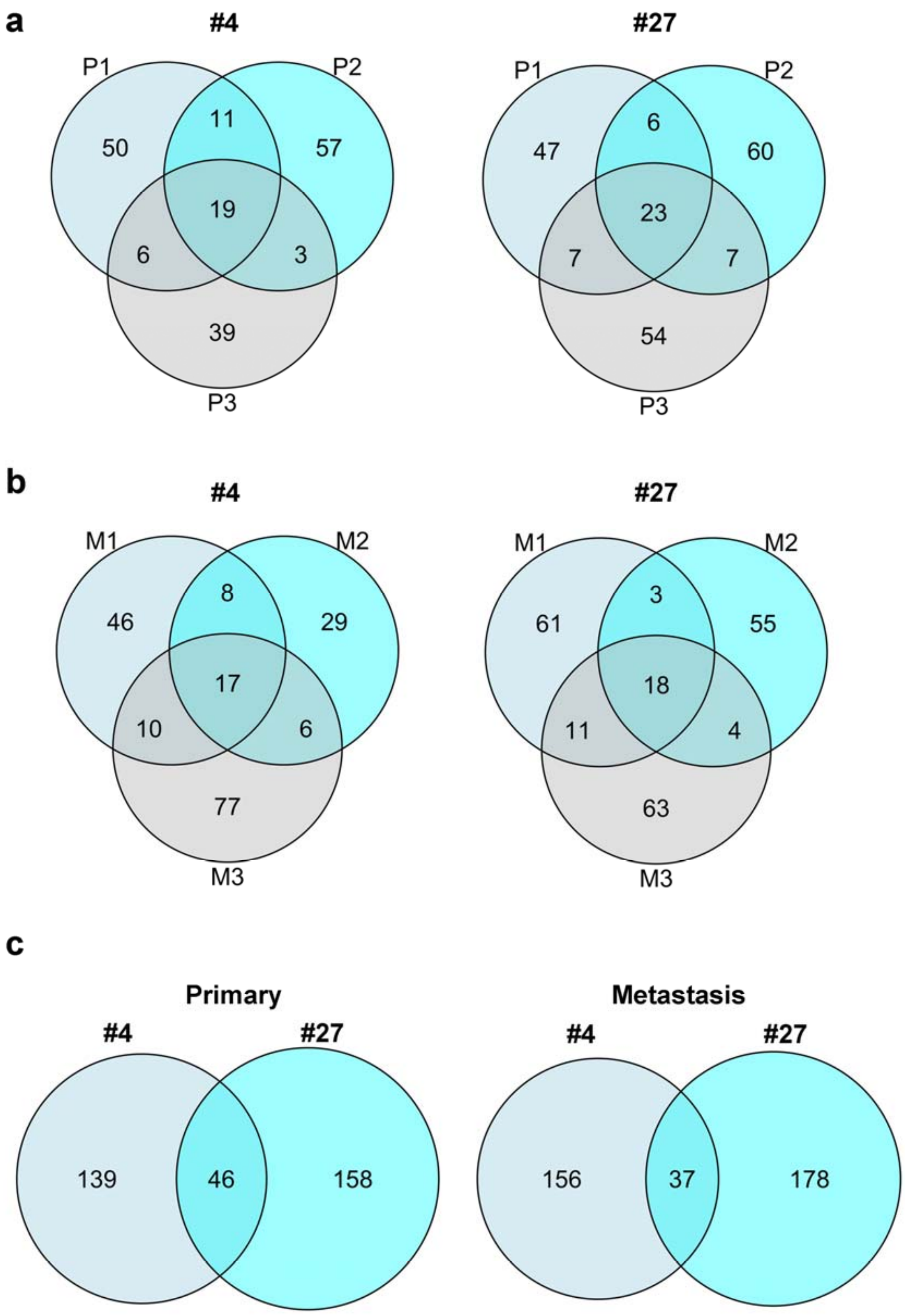

Supplementary Figure 7 Intra- and inter-tumor heterogeneity in mammary tumors driven by somatic *Trp53R245W* mutations, as revealed by multi-region exon sequencing.

a. Venn diagrams depicting the overlap of the identified mutations among three physically separated regions of primary tumors (P1-P3) from mouse #4 and #27.
b. Venn diagrams depicting the overlap of the identified mutations among three metastatic clones (M1-M3) in mouse #4 and #27.
c. Venn diagrams depicting the overlap of the identified mutations between #4 and #27 primary tumors (P) and metastases (M).

Supplementary Fig. 8

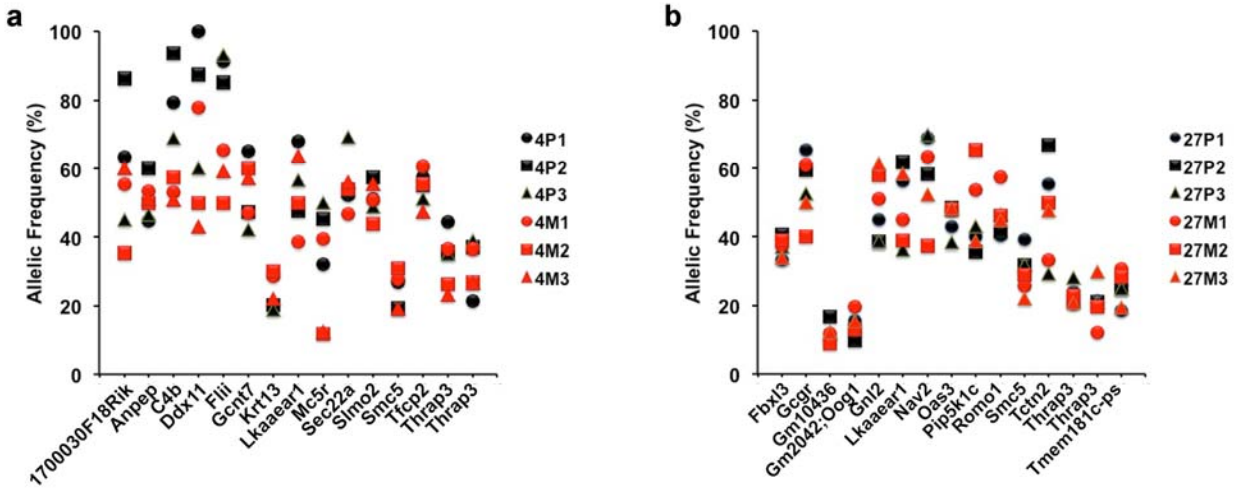

Supplementary Figure 8 Allelic frequencies of gene alterations that are present in all six samples (P1-P3 and M1-M3) in mouse #4 (a) and #27 (b).

Supplementary Fig. 9

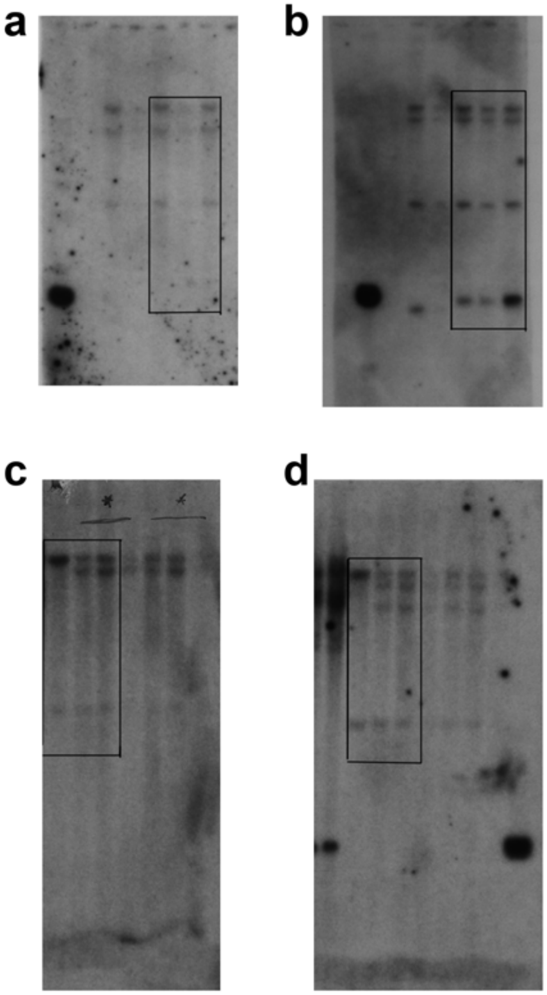

Supplementary Figure 9 Southern blots of cropped blots in the Supplementary Figure1.
a and b. Southern blots of cropped blots in the Supplementary Figure 1c.
c and d. Southern blots of cropped blots in the Supplementary Figure 1d.

Supplementary Fig. 10

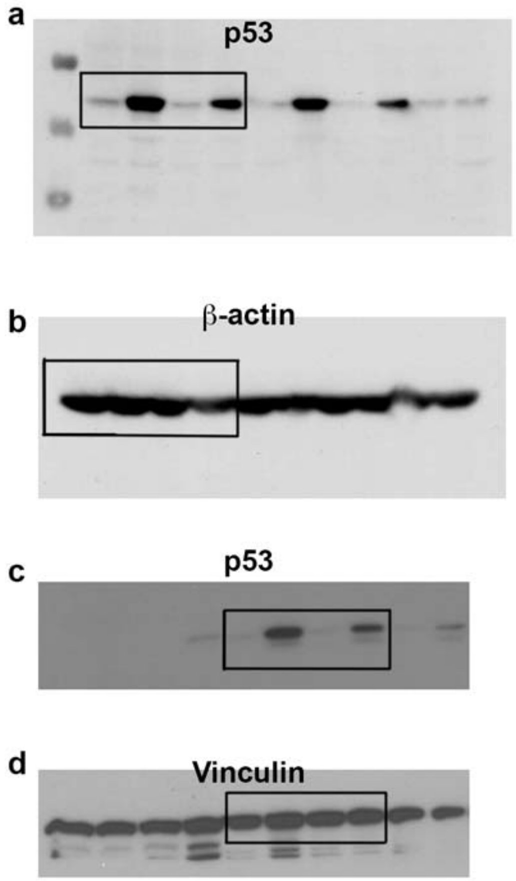

Supplementary Figure 10 Immunoblots of cropped blots in Figure 1b.
a and b. immunoblots of cropped blots for characterizing the *Trp53*$^{wm\text{-}R172H}$ allele as shown in Figure 1b.
c and d. immunoblots of cropped blots for characterizing the *Trp53*$^{wm\text{-}R245W}$ allele as shown in Figure 1b.
