# Supplementary material for: Somatic Trp53 mutations differentially drive breast cancer and evolution of metastases
Source: Nat Commun. 2018 Sep 27;9:3953. doi: 10.1038/s41467-018-06146-9 (PMC6160420; doi:10.1038/s41467-018-06146-9)
Supplement: Supplementary file 2 — Description of Additional Supplementary Files [file 41467_2018_6146_MOESM2_ESM.pdf]

Supplementary Data 1

Spreadsheet of genes with various alterations identified in any of the three physically separated regions of primary tumors (P1-P3) or associated metastatic clones (M1-M3) from each of two Trp53wm-R245W/+ mice (#4 and #27), subjected to high dose Ad-Cre intraductal injection. Column A, names of genes with alterations that have been identified (indicated by X in Columns B-M) in at least one region of P1-P3 or M1-M3 from #4 or #27 mouse. Columns N-Y, specific types of gene alterations. INS, insertion; DEL, deletion; NS, non-synonymous substitution; ncRNA_exonic, alteration in non-coding RNA exons; Splicing, alteration in splice junction; MultipleAlt, multiple alterations.
